# Supplementary figures and images for: Association between iscR-based phylogeny, serovars and potential virulence markers of Haemophilus parasuis
Source: PeerJ. 2019 May 14;7:e6950. doi: 10.7717/peerj.6950 (PMC6524630; doi:10.7717/peerj.6950)

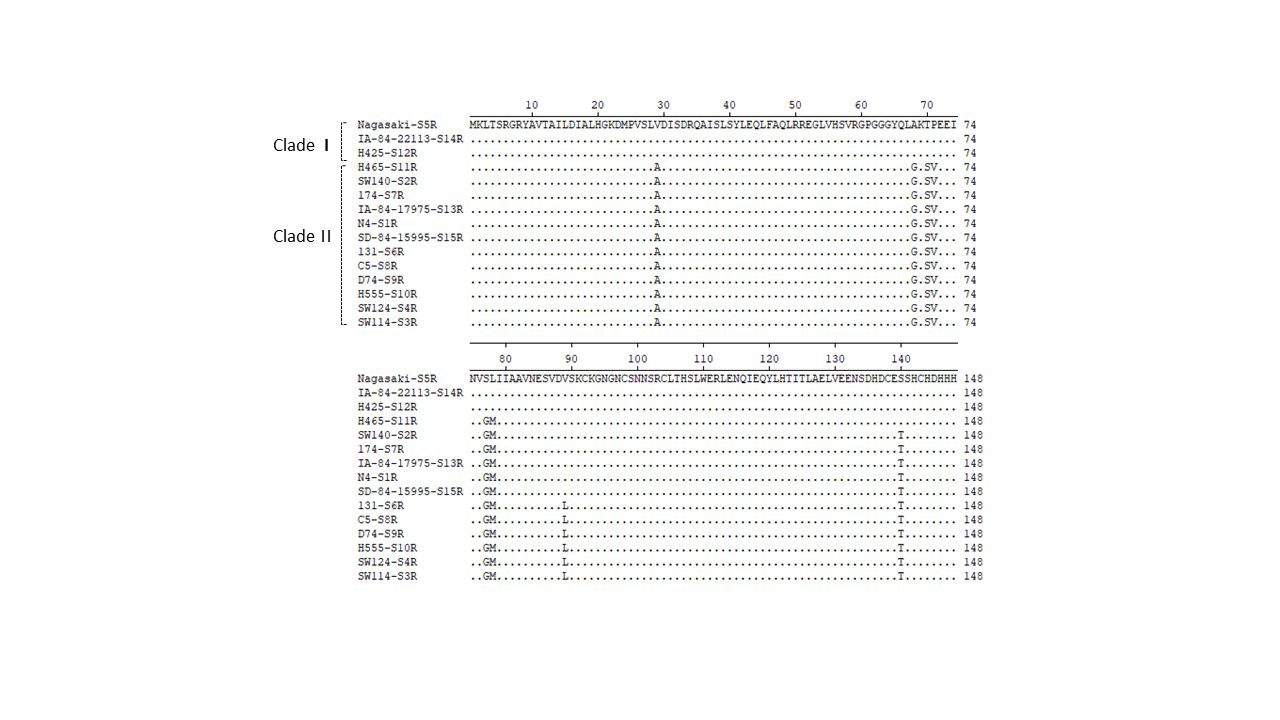

Supplement: Supplemental Information 1 — IscR sequences of 15 reference strains originated from a previous study (28). Sequence name includes information on both name (e.g., Nagasaki) and serovar (e.g., “S5R” is short for serovar 5 reference strain) of the strain. IscR sequence of Nagasaki-S5R were used as markers, and residues that match Nagasaki-S5R exactly were hidden (as “ . ”). The clade distribution of each sequence type was indicated on the left. [file peerj-07-6950-s001.png]
